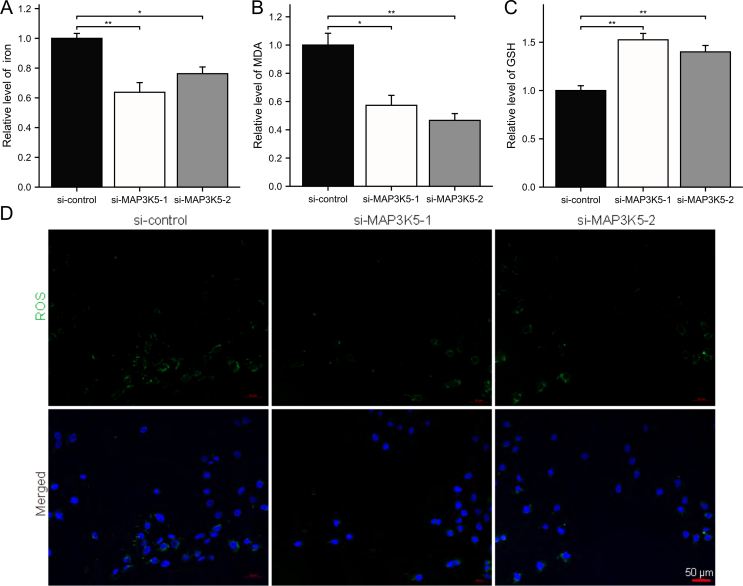# Multi-omics analysis to identify CBR3-AS1-hsa-miR-145-5p-MAP3K5 pathway as a ferroptosis-related ceRNA network in benign prostatic hyperplasia

**DOI:** 10.1016/j.gendis.2023.101184

**Published:** 2023-11-28

**Authors:** Liang Zhou, Youyou Li, Jiaren Li, Hanyu Yao, Jin Huang, Cheng Li, Long Wang

**Affiliations:** Department of Urology, The Third Xiangya Hospital of Central South University, Changsha, Hunan 410013, China

In aging males, benign prostatic hyperplasia (BPH) is a chronic pathological process that primarily causes lower urinary tract symptoms. According to histopathology, BPH is caused by an imbalance between cell death and proliferation.^1^ However, the exact pathophysiology remains unclear. Ferroptosis is a newly discovered form of programmed cell death. The cell morphology is characterized primarily by the reduction or disappearance of mitochondrial cristae and mitochondrial shrinkage.^2^^,^^3^ Competing endogenous RNAs (ceRNAs) are important regulators of many biological processes and have been involved in various diseases.^4^

This study integrated transcriptomics and metabolomics to analyze five BPH tissues and five control (normal) prostate tissues. The volcano plots of RNA and metabolite expression changes in human BPH tissue and normal prostate tissue are generated (Fig. S1A–C). Furthermore, the thresholds for identifying differentially expressed genes (DEGs) were set at adjusted *P*-value < 0.05 and |log fold change| > 1. The thresholds for identifying differentially expressed metabolites (DEMs) were set at *P*-value < 0.05 and variable importance in projection > 1. A total of 1861 DEGs (1086 up-regulated genes and 765 down-regulated genes) and 137 DEMs (71 positive and 66 negative ions) were identified. Heatmaps are then produced (Fig. S1D–F). Gene set enrichment analysis (GSEA), Gene Ontology (GO), and Kyoto Encyclopedia of Genes and Genomes (KEGG) analyses were conducted using R software to analyze the potential biological functions of DEGs. The significant GO terms were found to be involved in muscle system process and muscle contraction (biological processes); contractile fiber par and contractile fiber (cellular components); actin binding and metal ion transmembrane transporter activity (molecular functions) (Fig. S2A–E). In the KEGG pathway analysis, DEGs were primarily involved in the pathway associated with ferroptosis (Fig. S3A–E). The GSEA result indicated that the significantly enriched genes were involved in hypertrophic cardiomyopathy and dilated cardiomyopathy. The GSEA of DEGs is shown in Figure S4. The findings indicate that transcriptional patterns were altered after BPH, resulting in the activation of ferroptosis-related pathways. The DEMs identified above were imported into the Metaboanalyst website for enrichment and pathway analyses to examine metabolic pathways in BPH further. Enrichment (Fig. S5A, B) and pathway (Fig. S5C, D) analyses showed that the onset of BPH was primarily associated with glutathione metabolism, glutamate metabolism, d-glutamine metabolism, and d-glutamate metabolism. These metabolisms were closely related to ferroptosis. An integrative analysis integrating DEGs and DEMs from transcriptomic and metabolomic data was performed to explore how transcriptional and metabolic mechanisms regulate BPH. Significant enrichment in ferroptosis-related pathways was observed, including dilated cardiomyopathy, hypertrophic cardiomyopathy, MAPK signaling pathway, and PI3K-Akt signaling pathway (Fig. S5E, F). Additionally, 32 differentially expressed ferroptosis-related genes (DEFRGs) were identified (Fig. S6A). The 32 DEFRGs are shown in a heatmap (Fig. S6B). Furthermore, CytoHubba and STRING were used to further analyze the protein–protein interaction network (Fig. S6C). The results demonstrated five key DEFRGs (Fig. S6D). Moreover, a complex protein–protein interaction network was constructed for five key genes (Fig. S6E). DEFRGs were classified as ferroptosis drivers, suppressors, and markers (Fig. S6F and Table S1). The oxidative stress, secondary lysosome, oxidoreductase activity, and acting on NAD(P)H were associated with significant GO-enriched terms. In the KEGG enrichment analysis, DEFRGs were primarily involved in fluid shear stress and atherosclerosis, non-alcoholic fatty liver disease, 2-oxocarboxylic acid metabolism, and protein processing in the endoplasmic reticulum. The GO and KEGG pathway analyses of DEFRGs are shown in Figure S7A–E. To validate key DEFRGs, the expression of five key DEFRGs in our specimens was measured. Results of microarrays in prostate tissue samples are similar (Fig. S8A, B), where the expression levels of AKR1C2 and TXNRD1 were significantly lower, and MAP3K5 expression level was significantly higher in BPH samples (Fig. S8C). AKR1C1 and NQO1 expression were not significantly different. Overall, in 40 patients, MAP3K5 mRNA expression level was significantly positively associated with age (*r* = 0.324, *P* = 0.041), IPSS-S (*r* = 0.391, *P* = 0.013), and total IPSS (*r* = 0.347, *P* = 0.028), but not with IPSS-V (Fig. S8D–F and Table S2). However, no significant association was observed between AKR1C2 and TXNRD1 mRNA levels and clinical data. According to immunofluorescence staining, MAP3K5 was primarily localized in epithelium (Fig. S8G). To investigate the impact of MAP3K5 on ferroptosis in prostate cells, intracellular iron, the lipid peroxidation product malondialdehyde, and the substrate glutathione were assessed following MAP3K5 knockdown. It was observed that MAP3K5 silencing led to a reduction in cellular iron content (Fig. S9A) and malondialdehyde levels (Fig. S9B), along with an elevation in glutathione levels (Fig. S9C) within the prostate cells (BPH-1). Simultaneously, intracellular reactive oxygen species levels were reduced (Fig. S9D).

TargetScan, miRWalk, and miRDB were searched to predict MAP3K5 upstream miRNAs (Fig. 1A). Finally, 17 miRNAs were potentially found to target MAP3K5 (Fig. 1B). Based on the study data, hsa-miR-145-5p was identified for further investigation (Fig. 1C). BPH samples exhibited significantly lower levels of 145-5p expression than normal prostate samples, which was consistent with the results obtained from analyses of prostate tissue mRNA microarrays (Fig. 1D, E). A total of 42 lncRNAs were predicted to bind to the hsa-miR-145-5p using the miRNet database (Fig. 1F). Together with our transcriptome data, one lncRNA (CBR3-AS1) was identified (Fig. 1G). BPH samples exhibited significantly higher levels of CBR3-AS1 expression than normal prostate samples, consistent with the transcriptome data (Fig. 1H, I). Finally, it has been found that CBR3-AS1-hsa-miR-145-5p-MAP3K5 served as a potential pathway for BPH. Additionally, BPH drug candidates were screened using MAP3K5. Based on the combined score, the top 10 candidate drugs were identified (Fig. 1J and Table S3). Following that, the CBR3-AS1-hsa-miR-145-5p-MAP3K5 pathway was identified by combining transcriptome sequencing data and ceRNA target gene prediction results (Fig. 1K). Recently, multi-omics approaches have been extensively used for investigating disease pathophysiology and identifying key therapeutic targets. Molecular alterations and disease pathogens can be discovered more comprehensively using multi-omics approaches than traditional hypothesis-driven approaches.^5^ In this study, human prostate tissue was used for transcriptomic and metabolomic analysis to uncover potential therapeutic targets in BPH.

**Figure 1:**
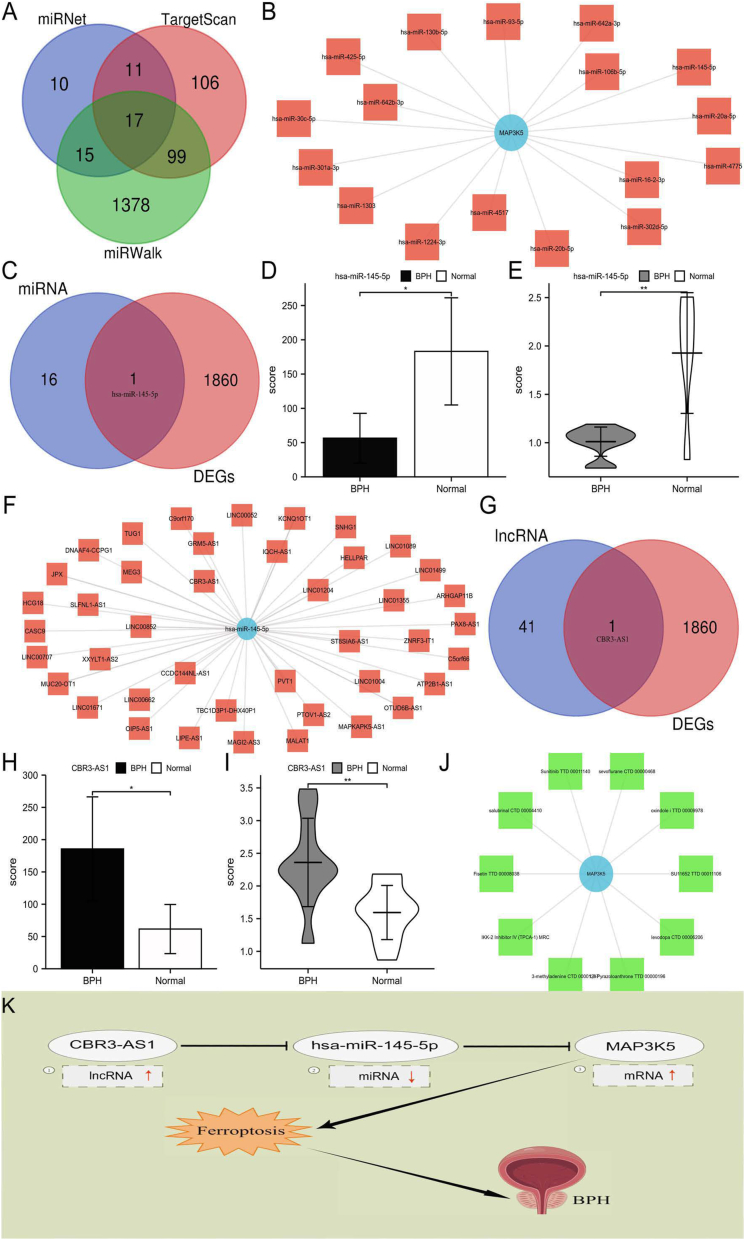
CBR3-AS1-hsa-miR-145-5p-MAP3K5 pathway identification. **(A)** Identification of upstream potential miRNAs. **(B)** The miRNA-MAP3K5 network established. **(C)** Co-expression of miRNAs. **(D, E)** The levels of hsa-miR-145-5p expressions in transcriptomics dates (D) and qRT-PCR analysis (E). **(F)** LncRNAs-hsa-miR-145-5p network. **(G)** Co-expression of lncRNAs. **(H, I)** The levels of CBR3-AS expressions in transcriptomics dates (H) and qRT-PCR analysis (I). **(J)** Combining MAP3K5 with the top 10 candidate drugs. **(K)** Model of the CBR3-AS1-hsa-miR-145-5p-MAP3K5 pathway.

In conclusion, this study demonstrated that ferroptosis might have a key role in BPH by combining transcriptomic and untargeted metabolomic analysis. As a ferroptosis-related ceRNA network, the CBR3-AS1-hsa-miR-145-5p-MAP3K5 pathway was found in BPH, providing a potential therapeutic target for BPH treatment.

## Ethics declaration

The study data were approved by the Ethics Committee of the Third Xiangya Hospital of Central South University (approval number: 22239).

## Funding

This work was supported by the 10.13039/501100001809National Natural Science Foundation of China (No. 82370779) and the Wisdom Accumulation and Talent Cultivation Project of the Third 10.13039/501100011790Xiangya Hospital of 10.13039/501100002822Central South University (No. 20210312).

## Data availability

Data supporting the findings of this study can be obtained from the corresponding author.

## Conflict of interests

The authors reported no potential conflict of interests.

## Figures and Tables

**figs1:**
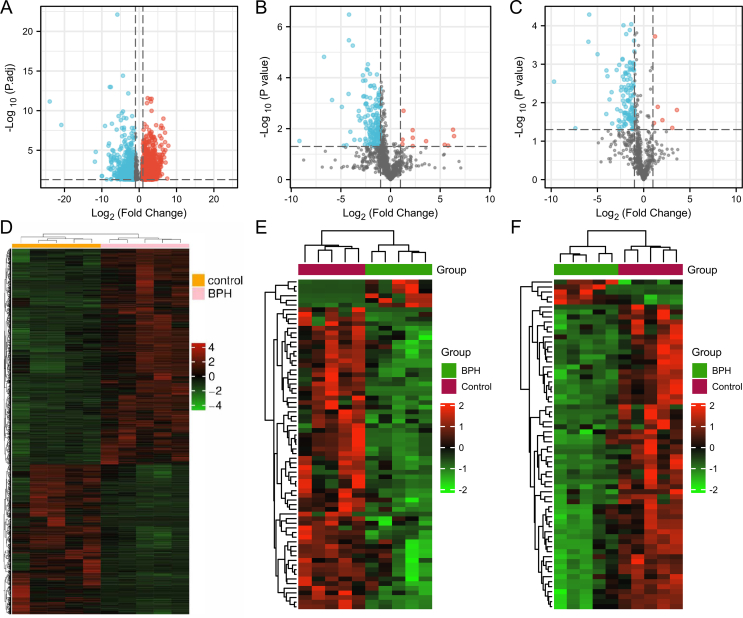


**figs2:**
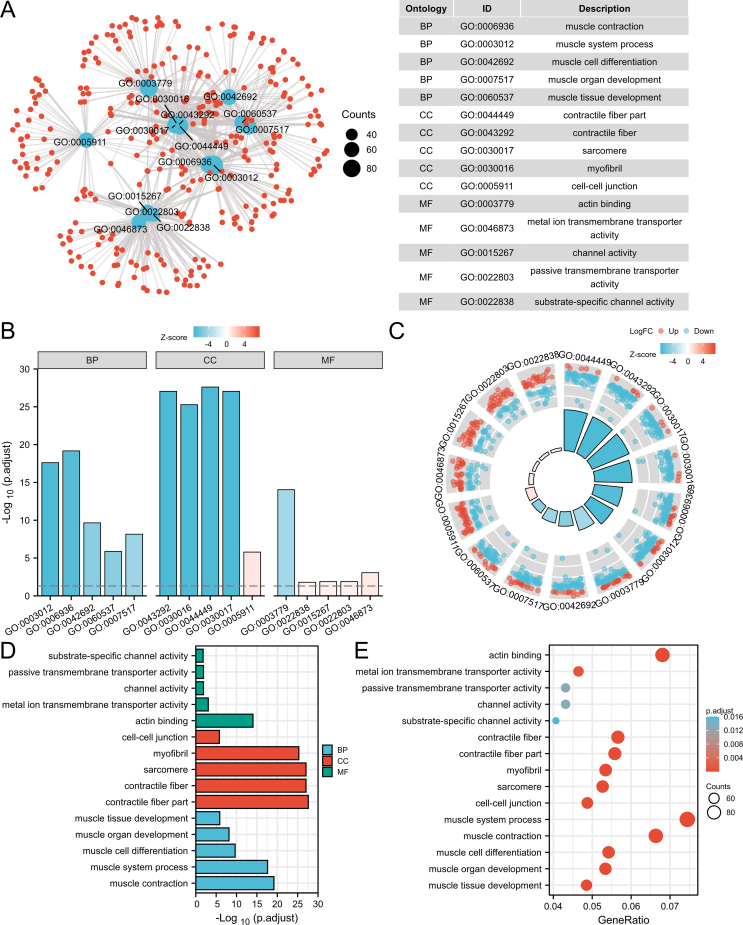


**figs3:**
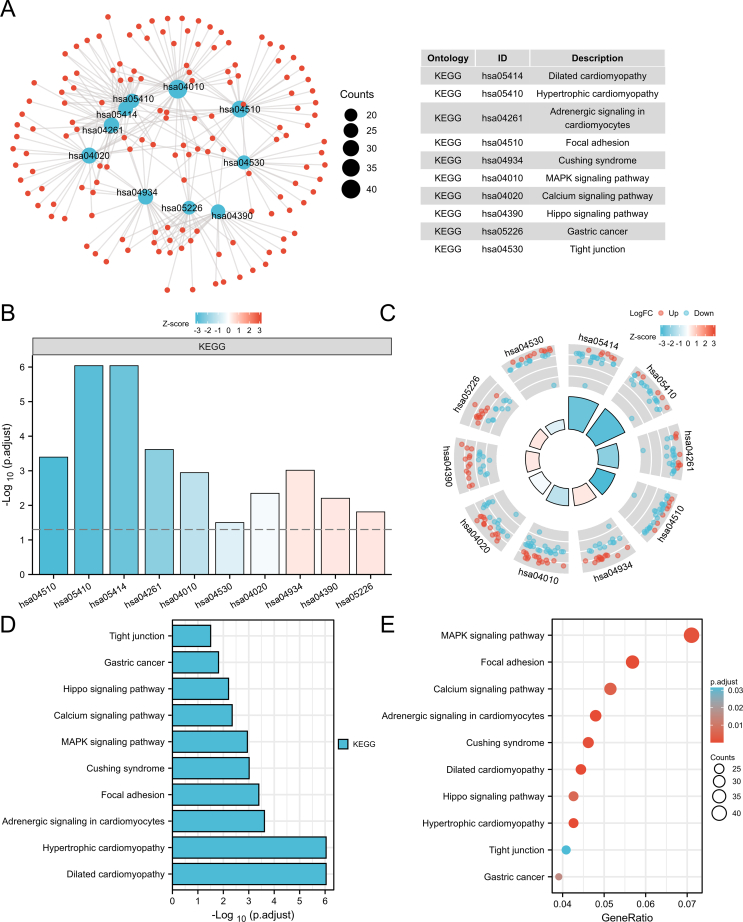


**figs4:**
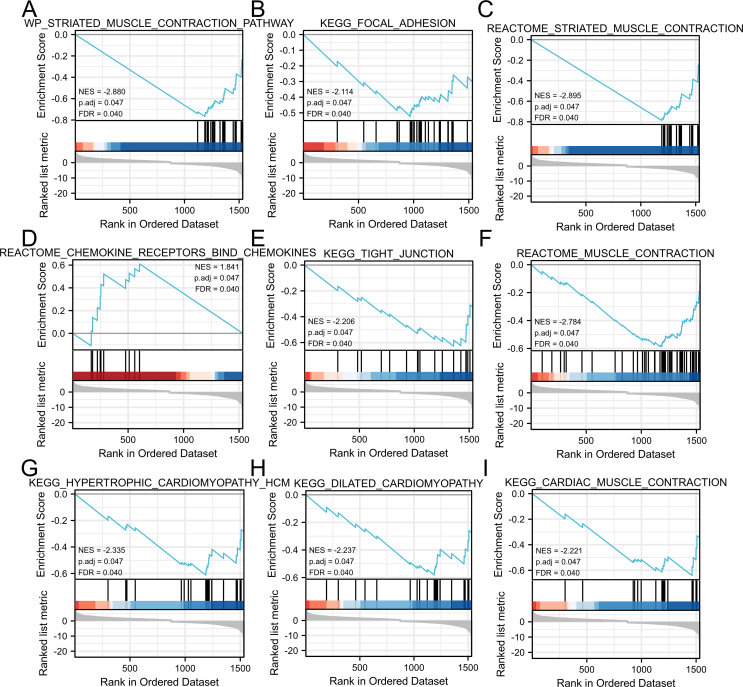


**figs5:**
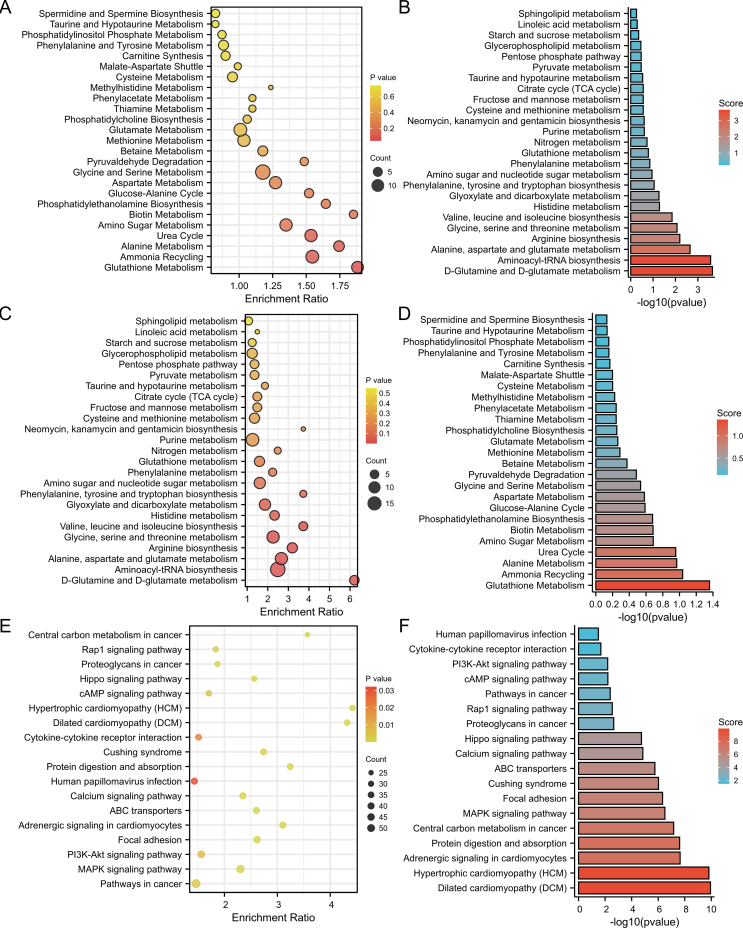


**figs6:**
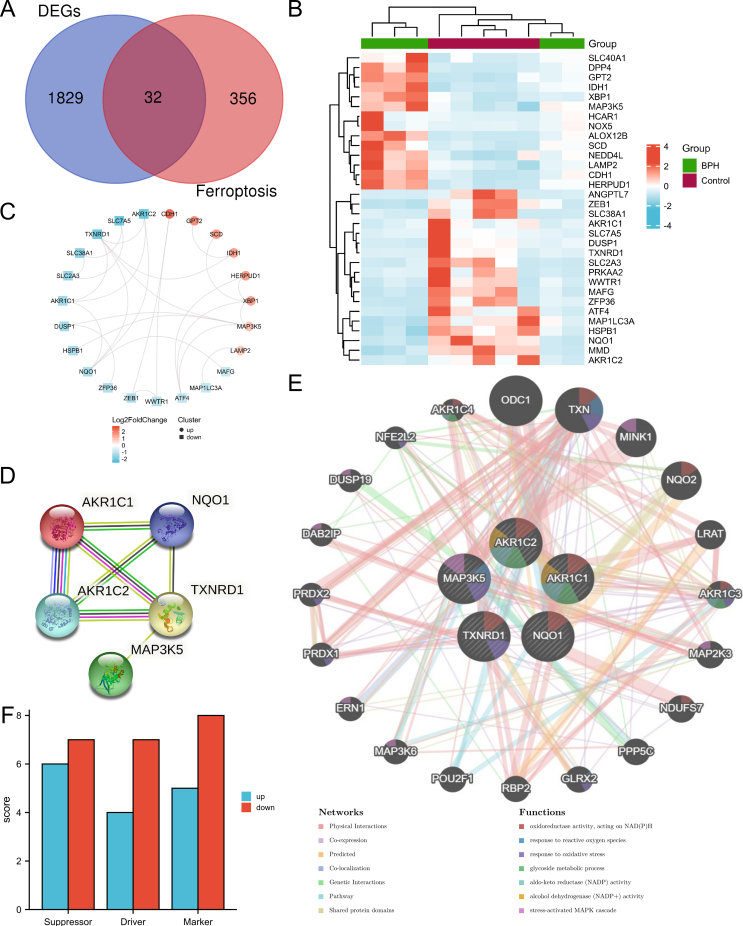


**figs7:**
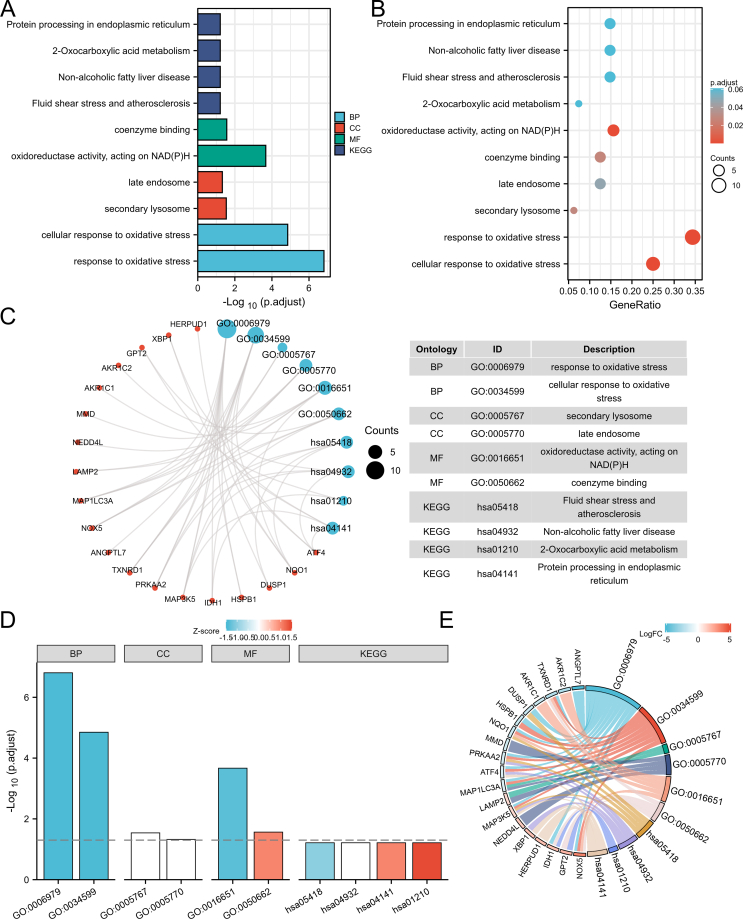


**figs8:**
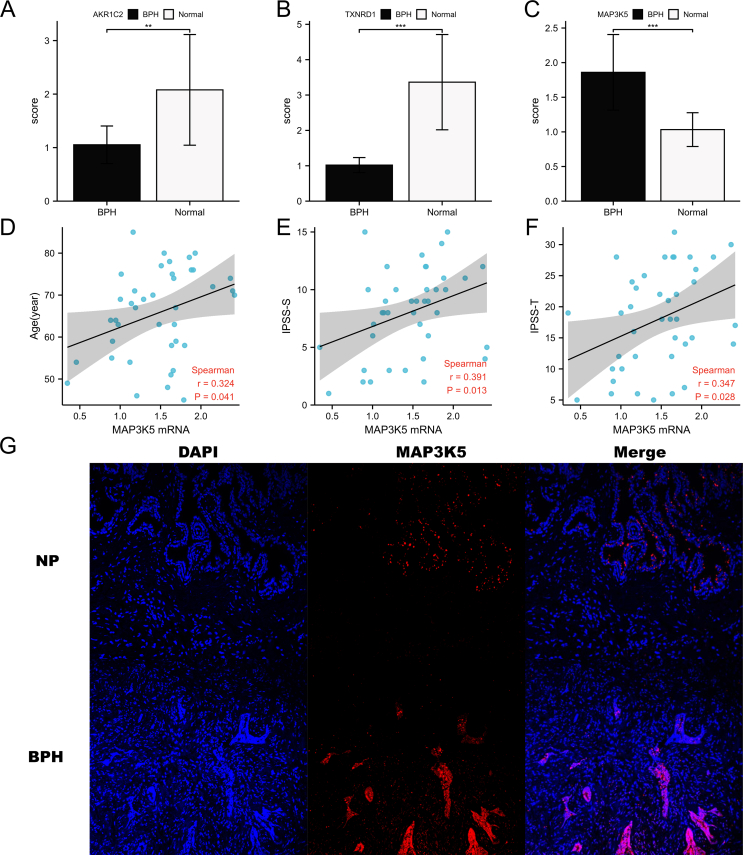


**figs9:**