# Supplementary material for: Multi-omics analysis to identify CBR3-AS1-hsa-miR-145-5p-MAP3K5 pathway as a ferroptosis-related ceRNA network in benign prostatic hyperplasia
Source: Genes Dis. 2023 Nov 28;11(5):101184. doi: 10.1016/j.gendis.2023.101184 (PMC11176642; doi:10.1016/j.gendis.2023.101184)
Supplement: Multimedia component 1 [file mmc1.docx]

**Supplementary Methods**

**Methods**

**Samples collection**

The surgical specimens collected from patients with BPH were used to obtain BPH tissue samples. Because BPH often has no adjacent normal tissue due to its nature, normal prostate samples from patients with bladder cancer were used as control group. All specimens were confirmed by pathological examination at the Third Xiangya Hospital of Central South University.

**RNA-seq and untargeted metabolomics analysis**

The spectrophotometer NanoPhotometer® (IMPLEN, CA, USA) was utilized in this investigation to evaluate the purity of RNA. The RNA Nano 6000 Assay Kit of the Bioanalyzer 2100 system (Agilent Technologies, CA, USA) was used to verify RNA integrity. NEBNext® UltraTM RNA Library Prep Kit for Illumina® (NEB, USA) NEBNext® was used to generate sequencing libraries. The AMPure XP system (Beckman Coulter, Beverly, USA) was used to purify library fragments. PCR products were purified using the AMPure XP system, and library quality was assessed using the Agilent Bioanalyzer 2100. Untargeted metabolomics analysis was performed using a UHPLC (1290 Infinity LC, Agilent Technologies) coupled with a quadrupole time-of-flight (AB Sciex TripleTOF 6600).

**FRGs and data processing**

A total of 588 ferroptosis-related genes (FRGs) were identified using the Ferroptosis Database (FerrDb). The VIP value for each metabolite was calculated from the OPLS-DA model. With the R project, the differentially expressed genes (|logFC|> 1 and adjusted P-value <0.05) and differential metabolites (P-value <0.05 and VIP >1) between normal prostate tissue and BPH tissue were determined. In accordance with the negative binomial distribution, DESeq2 calculates differential expression from digital gene expression data.

**Enrichment analysis and PPI network**

Using R software, Gene Ontology (GO), Kyoto Encyclopedia of Gene and Genome (KEGG), and gene set enrichment analyses (GSEA) were carried out to determine the potential biological functions of DEGs and DEFRGs. Differentially expressed genes and differential metabolite data in RNA-Seq and metabolomics with human prostate tissue were analyzed using MetaboAnalyst. To obtain information about protein-protein interactions (PPIs), STRING (Search Tool for the Retrieval of Interacting Genes) was used to analyze gene data. Cytoscape could then be used to analyze PPI network information. Furthermore, analyzing PPI networks was done using CytoHubba. The key genes were analyzed using GeneMANIA.

**Establishment of ferroptosis-related ceRNA regulatory network**

Based on the significance of ferroptosis, a ferroptosis-related ceRNA network was constructed using the following steps: (1) CytoHubba was used to analyze the PPI network to discover the target genes and further screen through qRT-PCR and clinical correlation studies; (2) miRDB, TargetScan, and miRWalk were used to predict the potential miRNA of the target gene, and the potential miRNA was intersected with the DEGs to obtain the target miRNA; (3) miRNet (miRNet 2.0) was used to predict potential lncRNA, and the potential lncRNA was intersected with DEGs to obtain the target lncRNA; (4) lncRNA-miRNA-mRNA triple regulatory network was built by integrating lncRNA-miRNA pair and miRNA-mRNA pair.

**qRT -PCR analysis**

In this study, total RNA from BPH and normal prostate samples were extracted. VarioskanTM LUX multimode microplate reader (Thermo Scientific, USA) was used to evaluate the purity of RNA. All primers conducted using the SYBR (Bimake, USA). PCR conditions were optimized with gradient PCRs on the FTC-3000 (Funglyn Biotech, Canada). 2-^ΔΔct^ method was utilized to calculate the relative expression.

**Immunofluorescence assay**

We sectioned human prostate tissue into 5 mm thick slices. MAP3K5 antibody was incubated overnight with the primary antibody mixture. Secondary antibodies were labeled with Alexa Fluor® 594-labeled anti-Rabbit IgG (1:300) to visualize the localization of the two primary antibodies. Apotome 2.0 (ZEISS, Germany) was utilized to visualize the images. Information on the antibody is summarized.

**Statistical analysis**

Statistical analysis was performed using R software. Student’s t-test was used to determine the gene expression levels of our clinical samples. Spearman’s coefficient analysis tested the correlation between gene mRNA expression level and clinically relevant data of BPH patients. It was considered statistically significant if P <0.05.

**Supplemental figure legends**

**Figure S1** Differentially expressed genes and differential metabolites. **(A)** Volcano plot of transcriptome expression changes. **(B, C)** Volcano plots of metabolome expression changes in positive ion (B) and negative ion ionization mode (C). **(D)** Heatmaps of differentially expressed genes. **(E, F)** Heatmaps of differential metabolites in positive ion (E) and negative ion ionization mode (F).

**Figure S2** Gene Ontology (GO) enrichment analysis of differentially expressed genes.

**Figure S3** Kyoto Encyclopedia of Genes and Genomes (KEGG) pathway analysis of differentially expressed genes.

**Figure S4** Gene set enrichment analysis (GSEA) of differentially expressed genes. **(A–I)** GSEA indicated that the genes significantly enriched were involved in the striated muscle contraction pathway (A), focus adhesion (B), reactive striated muscle contraction (C), response chemokine receptor to chemokine binding (D), tight connection (E), reactive muscle contraction (F), hypertrophic cardiomyopathy (G), dilated cardiomyopathy (H), and cardiac muscle contraction (I).

**Figure S5** Metaboanalyst website analysis. **(A, B)** Enrichment analysis of differential metabolites. **(C, D)** Pathway analysis of differential metabolites. **(E, F)** Integrated analysis of DEGs and differential metabolites.

**Figure S6** Protein-protein interaction (PPI) network of differentially expressed ferroptosis-related genes (DEFRGs). **(A, B)** The 32 DEFRGs in prostate tissues (A) and their heatmap (B). **(C, D)** The 32 DEFRGs were analyzed for PPIs (C) and 5 key DEFRGs were extracted from the PPI network (D). **(E)** Analyses of the functions of the five key genes based on the PPI network. **(F)** Ferroptosis drivers, suppressors, and markers were classified as DEFRGs.

**Figure S7** Enrichment analysis of differentially expressed ferroptosis-related genes (DEFRGs). Analysis of DEFRGs between BPH and normal samples using Gene Ontology (GO) and Kyoto Encyclopedia of Genes and Genomes (KEGG) pathways enrichment.

**Figure S8** The expression and clinical correlation of key differentially expressed ferroptosis-related genes (DEFRGs). **(A, B)** The levels of AKR1C2 and TXNRD1 expressions. **(C–F)** The levels of MAP3K5 expression **(C)**, age **(D)**, storage score **(E)**, and total score **(F)** were positively correlated with MAP3K5 mRNA expression. **(G)** MAP3K5 was primarily localized in epithelium.

**Figure S9** The effects of MAP3K5 knockdown on ferroptosis and reactive oxygen species (ROS) levels in prostate cells. **(A–C)** The levels of iron (A), malondialdehyde (MDA) (B), and glutathione (GSH) (C) in BPH-1 cells were determined through colorimetric assay kits. **(D)** The accumulation of ROS was analyzed using the DCFH-DA fluorescent probe.
