# Supplementary material for: Multi-omics analysis to identify CBR3-AS1-hsa-miR-145-5p-MAP3K5 pathway as a ferroptosis-related ceRNA network in benign prostatic hyperplasia
Source: Genes Dis. 2023 Nov 28;11(5):101184. doi: 10.1016/j.gendis.2023.101184 (PMC11176642; doi:10.1016/j.gendis.2023.101184)
Supplement: Multimedia component 2 [file mmc2.docx]

**Table S1**: Differentially expressed ferroptosis-related genes (DEFRGs) were classified into ferroptosis drivers, suppressors, and markers.

|  | **Suppressor** | **Driver** | **Marker** |
| --- | --- | --- | --- |
| **UP** | HCAR1, CDH1, SCD, SLC40A1, NEDD4L, LAMP2 | ALOX12B, DPP4,  NOX5, IDH1 | GPT2, HERPUD1, XBP1,  SLC40A1, MAP3K5 |
| **DOWN** | ATF4, PRKAA2, ZFP36,  NQO1, HSPB1, AKR1C1,  AKR1C2 | MAP1LC3A, ATF4,  PRKAA2, WWTR1,  ZEB1, MMD, SLC38A1 | MAFG, ATF4, HSPB1,  DUSP1, SLC2A3, TXNRD1, SLC7A5, ANGPTL7 |
