# Supplementary material for: Multi-omics analysis to identify CBR3-AS1-hsa-miR-145-5p-MAP3K5 pathway as a ferroptosis-related ceRNA network in benign prostatic hyperplasia
Source: Genes Dis. 2023 Nov 28;11(5):101184. doi: 10.1016/j.gendis.2023.101184 (PMC11176642; doi:10.1016/j.gendis.2023.101184)
Supplement: Multimedia component 3 [file mmc3.docx]

**Table S2**: Clinical data and MAP3K5 mRNA expression.

| **Variable** | **Pearson correlation coefficient** | **p value** |
| --- | --- | --- |
| Age | 0.324 | 0.041 |
| BMI | −0.076 | 0.639 |
| IPSS-Total | 0.347 | 0.028 |
| IPSS-V | 0.209 | 0.196 |
| IPSS-S | 0.391 | 0.013 |
| QOL score | −0.140 | 0.387 |
| Total PSA (ng/ml) | 0.156 | 0.335 |
| TPV (ml) | 0.208 | 0.198 |
| Qmax (ml/s) | 0.005 | 0.977 |
| PVR (ml) | −0.056 | 0.731 |

BMI, body mass index; IPSS, International Prostate Symptom Score; V, voiding subscore; S, storage subscore; QOL, quality of life; PSA, prostate specific antigen; TPV, total prostate volume; Qmax, maximum flow rate; PVR, postvoid residual
