# Supplementary material for: Multi-omics analysis to identify CBR3-AS1-hsa-miR-145-5p-MAP3K5 pathway as a ferroptosis-related ceRNA network in benign prostatic hyperplasia
Source: Genes Dis. 2023 Nov 28;11(5):101184. doi: 10.1016/j.gendis.2023.101184 (PMC11176642; doi:10.1016/j.gendis.2023.101184)
Supplement: Multimedia component 4 [file mmc4.docx]

**Table 3**: The drug candidates combined with MAP3K5.

| **Drug name** | **P-value** | | **Combined score** |
| --- | --- | --- | --- |
| SU11652  TTD 00011106 | 7.00E-04 | | 145187.4 |
| oxindole i  TTD 00009978 | 7.50E-04 | | 143801.3 |
| sevoflurane  CTD 00000468 | 9.00E-04 | | 140136.5 |
| Sunitinib  TTD 00011140 | 1.00E-03 | | 138017.4 |
| salubrinal  CTD 00004410 | 0.001049976 | 137035.7 | |
| Fisetin  TTD 00008038 | 0.001049976 | 137035.7 | |
| IKK-2 Inhibitor IV (TPCA-1)  MRC | 0.001049976 | 137035.7 | |
| 3-methyladenine  CTD 00001217 | 0.001349972 | 131975 | |
| 1,9-Pyrazoloanthrone  TTD 00000196 | 0.001399971 | 131242.1 | |
| levodopa  CTD 00006206 | 0.001549969 | 129189.9 | |
